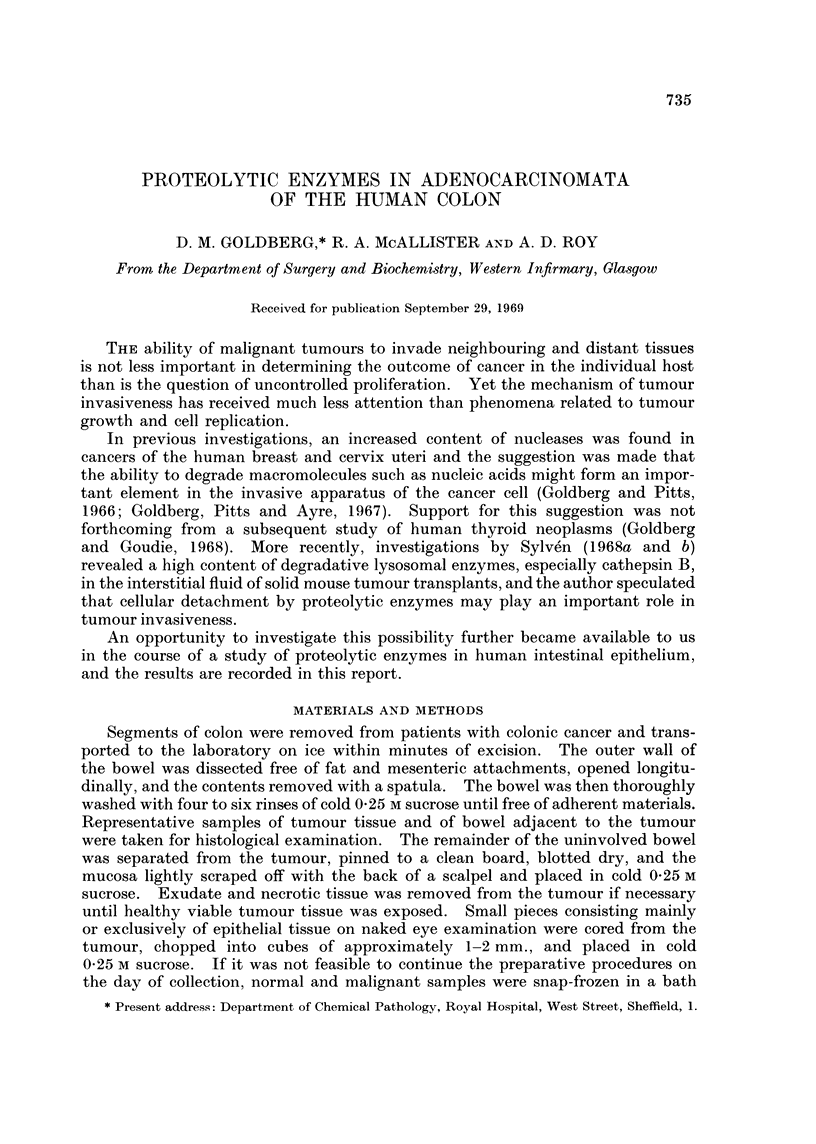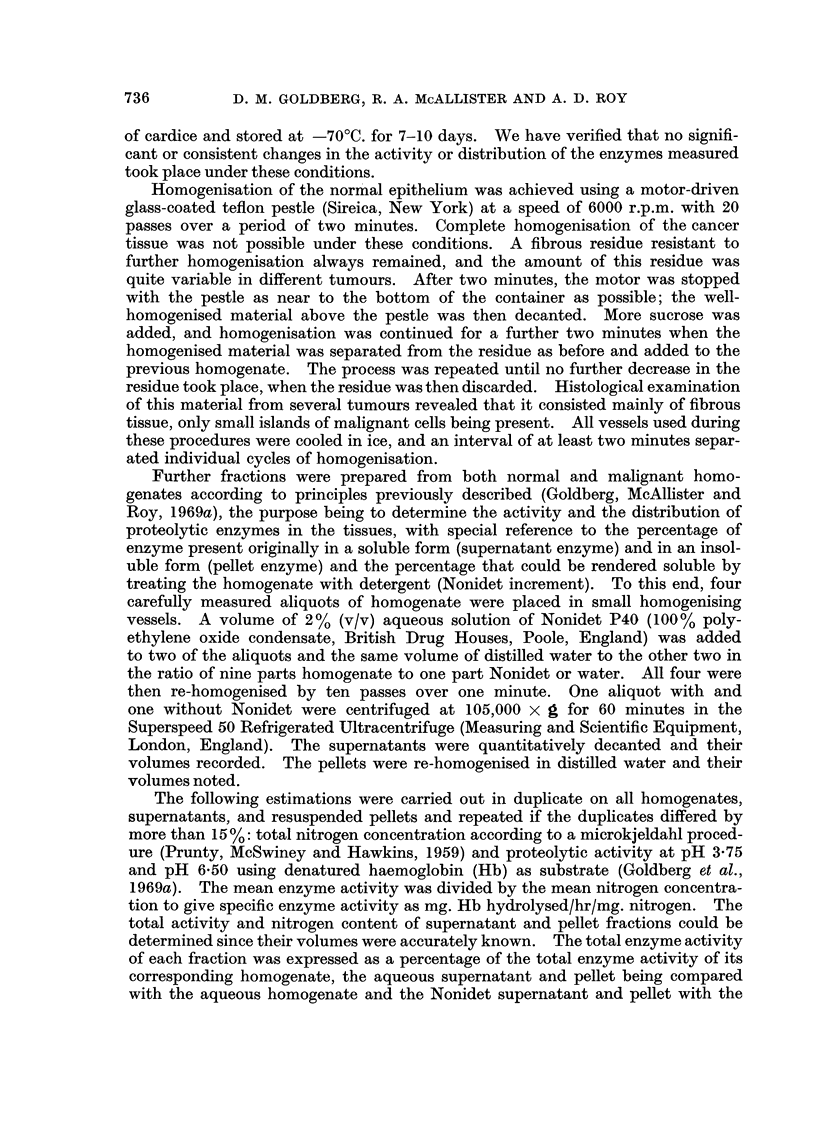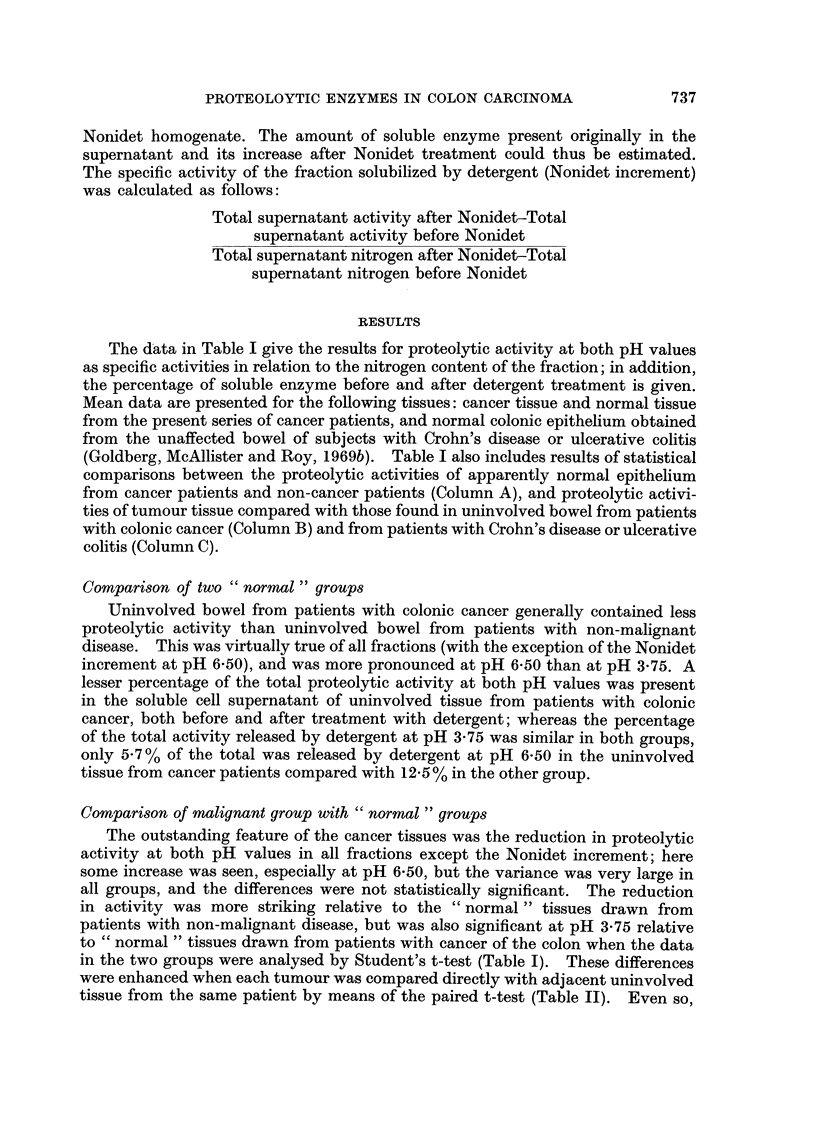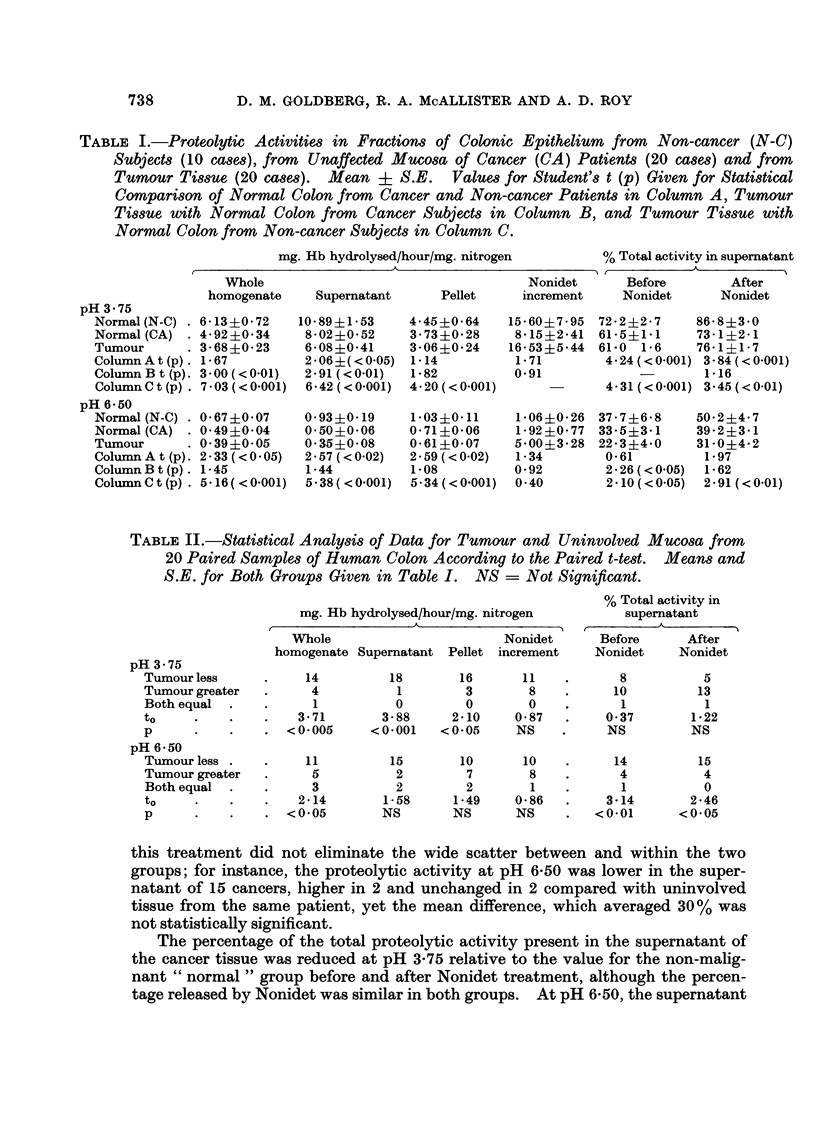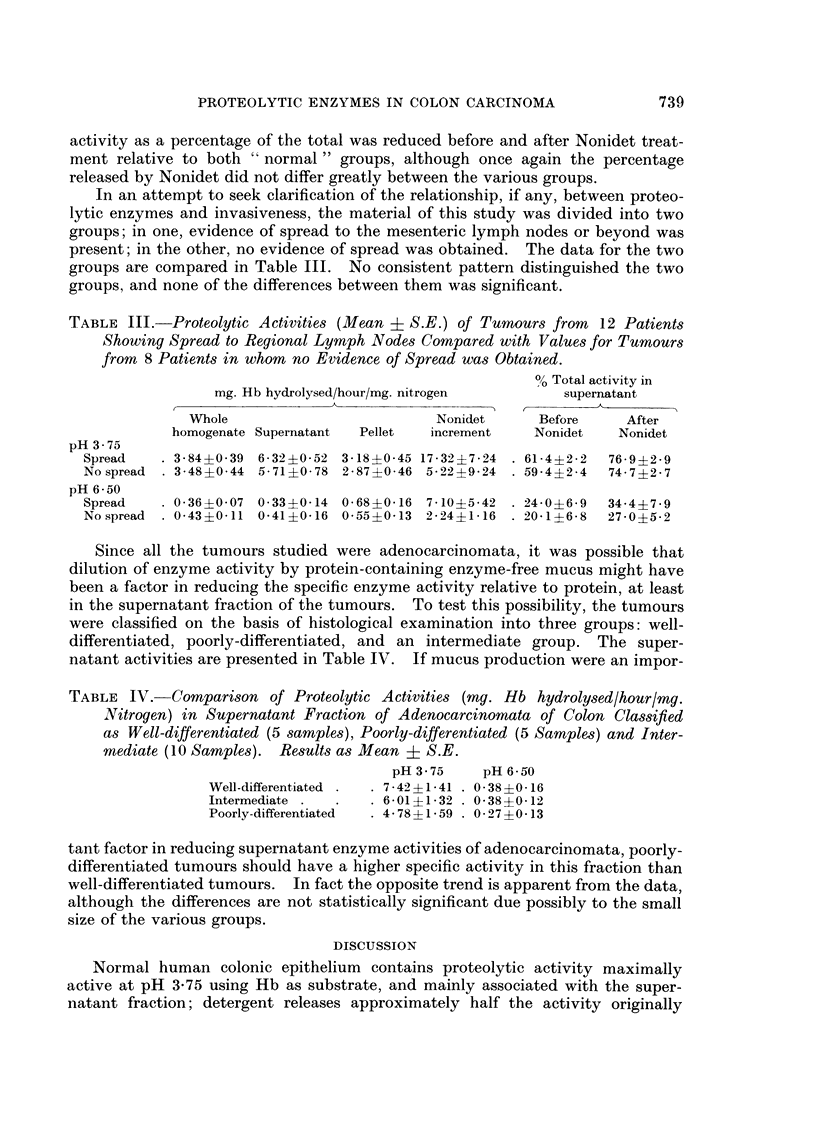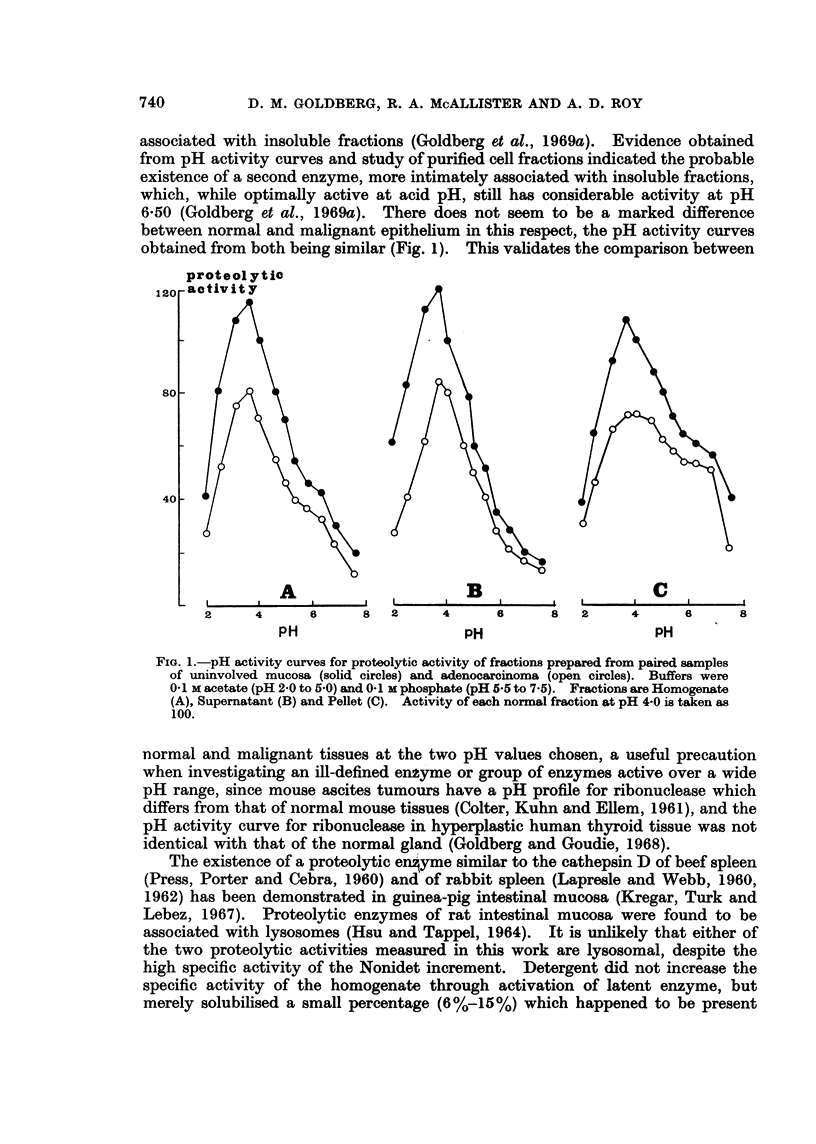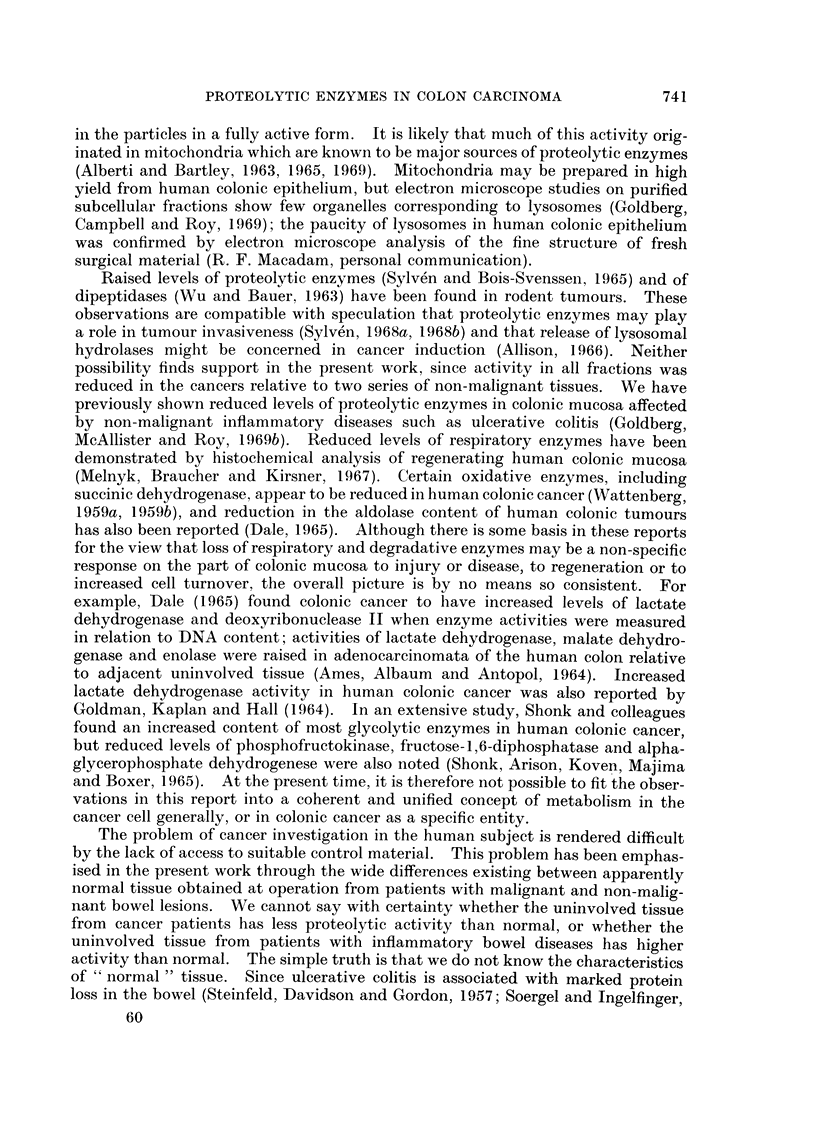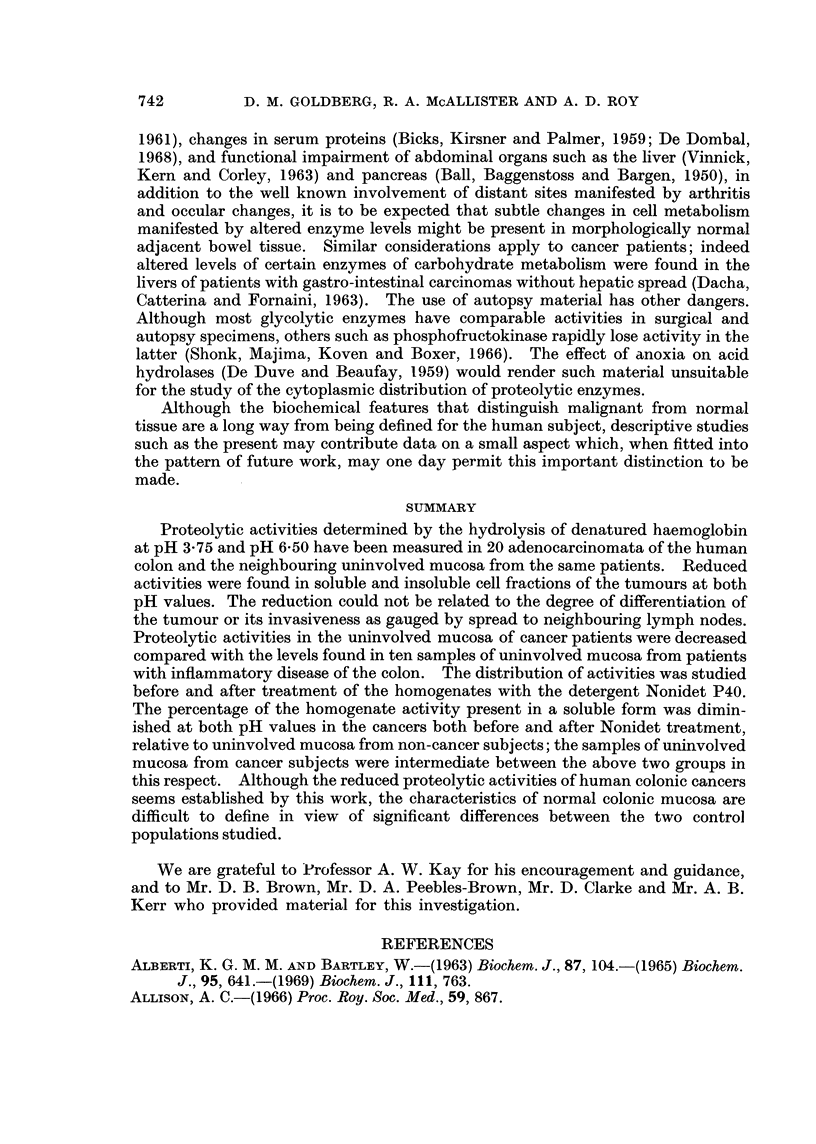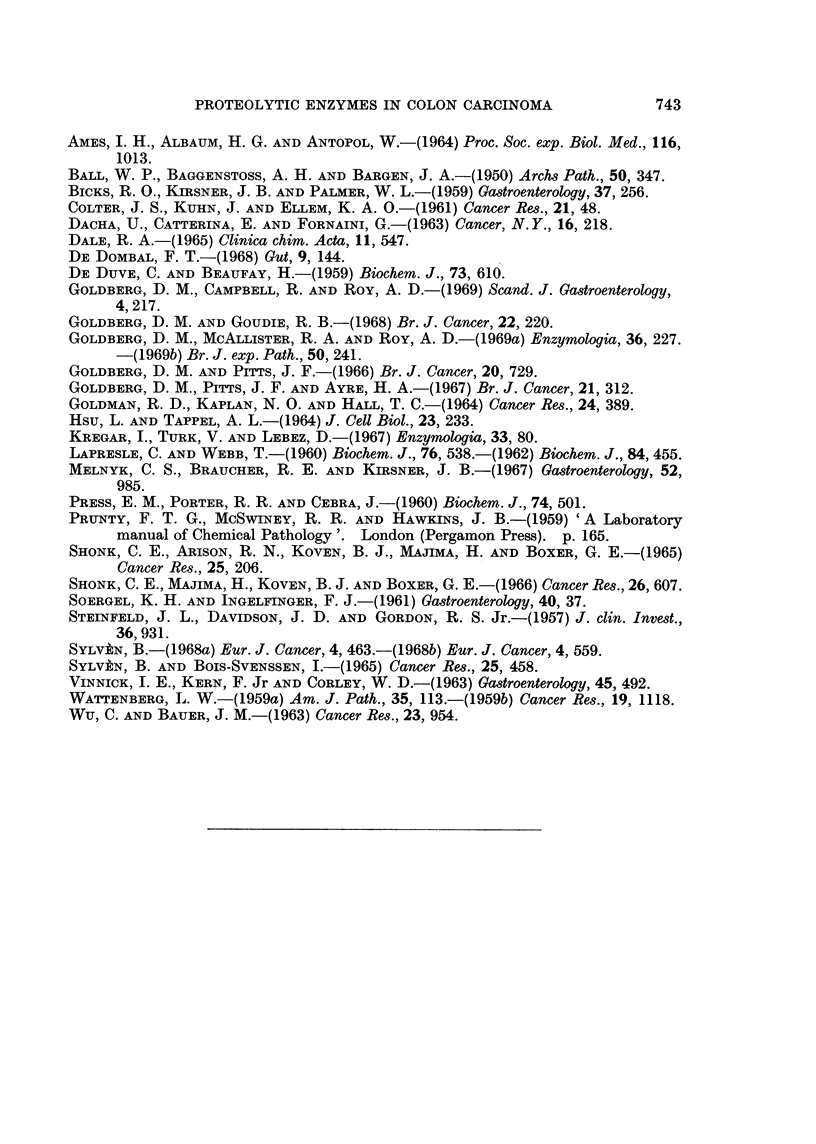# Proteolytic enzymes in adenocarcinomata of the human colon.

**DOI:** 10.1038/bjc.1969.90

**Published:** 1969-12

**Authors:** D. M. Goldberg, R. A. McAllister, A. D. Roy


### PROTEOLYTIC ENZYMES IN ADENOCARCINOMATA OF THE HUMAN COLON

D. M. GOLDBERG,* R. A. McALLISTER AND A. D. ROY

*From the Department of Surgery and Biochemistry, Western Infirmary, Glasgow*

Received for publication September 29, 1969

THE ability of malignant tumours to invade neighbouring and distant tissues is not less important in determining the outcome of cancer in the individual host than is the question of uncontrolled proliferation. Yet the mechanism of tumour invasiveness has received much less attention than phenomena related to tumour growth and cell replication.

In previous investigations, an increased content of nucleases was found in cancers of the human breast and cervix uteri and the suggestion was made that the ability to degrade macromolecules such as nucleic acids might form an important element in the invasive apparatus of the cancer cell (Goldberg and Pitts, 1966; Goldberg, Pitts and Ayre, 1967). Support for this suggestion was not forthcoming from a subsequent study of human thyroid neoplasms (Goldberg and Goudie, 1968). More recently, investigations by Sylvén (1968*a* and *b*) revealed a high content of degradative lysosomal enzymes, especially cathepsin B, in the interstitial fluid of solid mouse tumour transplants, and the author speculated that cellular detachment by proteolytic enzymes may play an important role in tumour invasiveness.

An opportunity to investigate this possibility further became available to us in the course of a study of proteolytic enzymes in human intestinal epithelium, and the results are recorded in this report.

### MATERIALS AND METHODS

Segments of colon were removed from patients with colonic cancer and transported to the laboratory on ice within minutes of excision. The outer wall of the bowel was dissected free of fat and mesenteric attachments, opened longitudinally, and the contents removed with a spatula. The bowel was then thoroughly washed with four to six rinses of cold 0·25 M sucrose until free of adherent materials. Representative samples of tumour tissue and of bowel adjacent to the tumour were taken for histological examination. The remainder of the uninvolved bowel was separated from the tumour, pinned to a clean board, blotted dry, and the mucosa lightly scraped off with the back of a scalpel and placed in cold 0·25 M sucrose. Exudate and necrotic tissue was removed from the tumour if necessary until healthy viable tumour tissue was exposed. Small pieces consisting mainly or exclusively of epithelial tissue on naked eye examination were cored from the tumour, chopped into cubes of approximately 1–2 mm., and placed in cold 0·25 M sucrose. If it was not feasible to continue the preparative procedures on the day of collection, normal and malignant samples were snap-frozen in a bath

* Present address: Department of Chemical Pathology, Royal Hospital, West Street, Sheffield, 1.

of cardice and stored at —70°C. for 7–10 days. We have verified that no significant or consistent changes in the activity or distribution of the enzymes measured took place under these conditions.

Homogenisation of the normal epithelium was achieved using a motor-driven glass-coated teflon pestle (Sireica, New York) at a speed of 6000 r.p.m. with 20 passes over a period of two minutes. Complete homogenisation of the cancer tissue was not possible under these conditions. A fibrous residue resistant to further homogenisation always remained, and the amount of this residue was quite variable in different tumours. After two minutes, the motor was stopped with the pestle as near to the bottom of the container as possible; the well-homogenised material above the pestle was then decanted. More sucrose was added, and homogenisation was continued for a further two minutes when the homogenised material was separated from the residue as before and added to the previous homogenate. The process was repeated until no further decrease in the residue took place, when the residue was then discarded. Histological examination of this material from several tumours revealed that it consisted mainly of fibrous tissue, only small islands of malignant cells being present. All vessels used during these procedures were cooled in ice, and an interval of at least two minutes separated individual cycles of homogenisation.

Further fractions were prepared from both normal and malignant homogenates according to principles previously described (Goldberg, McAllister and Roy, 1969*a*), the purpose being to determine the activity and the distribution of proteolytic enzymes in the tissues, with special reference to the percentage of enzyme present originally in a soluble form (supernatant enzyme) and in an insoluble form (pellet enzyme) and the percentage that could be rendered soluble by treating the homogenate with detergent (Nonidet increment). To this end, four carefully measured aliquots of homogenate were placed in small homogenising vessels. A volume of 2% (v/v) aqueous solution of Nonidet P40 (100% polyethylene oxide condensate, British Drug Houses, Poole, England) was added to two of the aliquots and the same volume of distilled water to the other two in the ratio of nine parts homogenate to one part Nonidet or water. All four were then re-homogenised by ten passes over one minute. One aliquot with and one without Nonidet were centrifuged at 105,000 × g for 60 minutes in the Superspeed 50 Refrigerated Ultracentrifuge (Measuring and Scientific Equipment, London, England). The supernatants were quantitatively decanted and their volumes recorded. The pellets were re-homogenised in distilled water and their volumes noted.

The following estimations were carried out in duplicate on all homogenates, supernatants, and resuspended pellets and repeated if the duplicates differed by more than 15%: total nitrogen concentration according to a microkjeldahl procedure (Prunty, McSwiney and Hawkins, 1959) and proteolytic activity at pH 3·75 and pH 6·50 using denatured haemoglobin (Hb) as substrate (Goldberg *et al.*, 1969*a*). The mean enzyme activity was divided by the mean nitrogen concentration to give specific enzyme activity as mg. Hb hydrolysed/hr/mg. nitrogen. The total activity and nitrogen content of supernatant and pellet fractions could be determined since their volumes were accurately known. The total enzyme activity of each fraction was expressed as a percentage of the total enzyme activity of its corresponding homogenate, the aqueous supernatant and pellet being compared with the aqueous homogenate and the Nonidet supernatant and pellet with the

Nonidet homogenate. The amount of soluble enzyme present originally in the supernatant and its increase after Nonidet treatment could thus be estimated. The specific activity of the fraction solubilized by detergent (Nonidet increment) was calculated as follows:

$$\frac{\text{Total supernatant activity after Nonidet–Total supernatant activity before Nonidet}}{\text{Total supernatant nitrogen after Nonidet–Total supernatant nitrogen before Nonidet}}$$

### RESULTS

The data in Table I give the results for proteolytic activity at both pH values as specific activities in relation to the nitrogen content of the fraction; in addition, the percentage of soluble enzyme before and after detergent treatment is given. Mean data are presented for the following tissues: cancer tissue and normal tissue from the present series of cancer patients, and normal colonic epithelium obtained from the unaffected bowel of subjects with Crohn's disease or ulcerative colitis (Goldberg, McAllister and Roy, 1969*b*). Table I also includes results of statistical comparisons between the proteolytic activities of apparently normal epithelium from cancer patients and non-cancer patients (Column A), and proteolytic activities of tumour tissue compared with those found in uninvolved bowel from patients with colonic cancer (Column B) and from patients with Crohn's disease or ulcerative colitis (Column C).

*Comparison of two " normal " groups*

Uninvolved bowel from patients with colonic cancer generally contained less proteolytic activity than uninvolved bowel from patients with non-malignant disease. This was virtually true of all fractions (with the exception of the Nonidet increment at pH 6·50), and was more pronounced at pH 6·50 than at pH 3·75. A lesser percentage of the total proteolytic activity at both pH values was present in the soluble cell supernatant of uninvolved tissue from patients with colonic cancer, both before and after treatment with detergent; whereas the percentage of the total activity released by detergent at pH 3·75 was similar in both groups, only 5·7% of the total was released by detergent at pH 6·50 in the uninvolved tissue from cancer patients compared with 12·5% in the other group.

*Comparison of malignant group with " normal " groups*

The outstanding feature of the cancer tissues was the reduction in proteolytic activity at both pH values in all fractions except the Nonidet increment; here some increase was seen, especially at pH 6·50, but the variance was very large in all groups, and the differences were not statistically significant. The reduction in activity was more striking relative to the " normal " tissues drawn from patients with non-malignant disease, but was also significant at pH 3·75 relative to " normal " tissues drawn from patients with cancer of the colon when the data in the two groups were analysed by Student's t-test (Table I). These differences were enhanced when each tumour was compared directly with adjacent uninvolved tissue from the same patient by means of the paired t-test (Table II). Even so,

TABLE I.—*Proteolytic Activities in Fractions of Colonic Epithelium from Non-cancer (N-C) Subjects (10 cases), from Unaffected Mucosa of Cancer (CA) Patients (20 cases) and from Tumour Tissue (20 cases). Mean ± S.E. Values for Student's t (p) Given for Statistical Comparison of Normal Colon from Cancer and Non-cancer Patients in Column A, Tumour Tissue with Normal Colon from Cancer Subjects in Column B, and Tumour Tissue with Normal Colon from Non-cancer Subjects in Column C.*

| | mg. Hb hydrolysed/hour/mg. nitrogen | | | | % Total activity in supernatant | |
|---|---|---|---|---|---|---|
| | Whole homogenate | Supernatant | Pellet | Nonidet increment | Before Nonidet | After Nonidet |
| pH 3·75 | | | | | | |
| Normal (N-C) | 6·13±0·72 | 10·89±1·53 | 4·45±0·64 | 15·60±7·95 | 72·2±2·7 | 86·8±3·0 |
| Normal (CA) | 4·92±0·34 | 8·02±0·52 | 3·73±0·28 | 8·15±2·41 | 61·5±1·1 | 73·1±2·1 |
| Tumour | 3·68±0·23 | 6·08±0·41 | 3·06±0·24 | 16·53±5·44 | 61·0  1·6 | 76·1±1·7 |
| Column A t (p) | 1·67 | 2·06±(<0·05) | 1·14 | 1·71 | 4·24 (<0·001) | 3·84 (<0·001) |
| Column B t (p) | 3·00 (<0·01) | 2·91 (<0·01) | 1·82 | 0·91 | — | 1·16 |
| Column C t (p) | 7·03 (<0·001) | 6·42 (<0·001) | 4·20 (<0·001) | — | 4·31 (<0·001) | 3·45 (<0·01) |
| pH 6·50 | | | | | | |
| Normal (N-C) | 0·67±0·07 | 0·93±0·19 | 1·03±0·11 | 1·06±0·26 | 37·7±6·8 | 50·2±4·7 |
| Normal (CA) | 0·49±0·04 | 0·50±0·06 | 0·71±0·06 | 1·92±0·77 | 33·5±3·1 | 39·2±3·1 |
| Tumour | 0·39±0·05 | 0·35±0·08 | 0·61±0·07 | 5·00±3·28 | 22·3±4·0 | 31·0±4·2 |
| Column A t (p) | 2·33 (<0·05) | 2·57 (<0·02) | 2·59 (<0·02) | 1·34 | 0·61 | 1·97 |
| Column B t (p) | 1·45 | 1·44 | 1·08 | 0·92 | 2·26 (<0·05) | 1·62 |
| Column C t (p) | 5·16 (<0·001) | 5·38 (<0·001) | 5·34 (<0·001) | 0·40 | 2·10 (<0·05) | 2·91 (<0·01) |

TABLE II.—*Statistical Analysis of Data for Tumour and Uninvolved Mucosa from 20 Paired Samples of Human Colon According to the Paired t-test. Means and S.E. for Both Groups Given in Table I. NS = Not Significant.*

| | mg. Hb hydrolysed/hour/mg. nitrogen | | | | % Total activity in supernatant | |
|---|---|---|---|---|---|---|
| | Whole homogenate | Supernatant | Pellet | Nonidet increment | Before Nonidet | After Nonidet |
| pH 3·75 | | | | | | |
| Tumour less | 14 | 18 | 16 | 11 | 8 | 5 |
| Tumour greater | 4 | 1 | 3 | 8 | 10 | 13 |
| Both equal | 1 | 0 | 0 | 0 | 1 | 1 |
| $t_0$ | 3·71 | 3·88 | 2·10 | 0·87 | 0·37 | 1·22 |
| p | <0·005 | <0·001 | <0·05 | NS | NS | NS |
| pH 6·50 | | | | | | |
| Tumour less | 11 | 15 | 10 | 10 | 14 | 15 |
| Tumour greater | 5 | 2 | 7 | 8 | 4 | 4 |
| Both equal | 3 | 2 | 2 | 1 | 1 | 0 |
| $t_0$ | 2·14 | 1·58 | 1·49 | 0·86 | 3·14 | 2·46 |
| p | <0·05 | NS | NS | NS | <0·01 | <0·05 |

this treatment did not eliminate the wide scatter between and within the two groups; for instance, the proteolytic activity at pH 6·50 was lower in the supernatant of 15 cancers, higher in 2 and unchanged in 2 compared with uninvolved tissue from the same patient, yet the mean difference, which averaged 30% was not statistically significant.

The percentage of the total proteolytic activity present in the supernatant of the cancer tissue was reduced at pH 3·75 relative to the value for the non-malignant " normal " group before and after Nonidet treatment, although the percentage released by Nonidet was similar in both groups. At pH 6·50, the supernatant activity as a percentage of the total was reduced before and after Nonidet treatment relative to both " normal " groups, although once again the percentage released by Nonidet did not differ greatly between the various groups.

In an attempt to seek clarification of the relationship, if any, between proteolytic enzymes and invasiveness, the material of this study was divided into two groups; in one, evidence of spread to the mesenteric lymph nodes or beyond was present; in the other, no evidence of spread was obtained. The data for the two groups are compared in Table III. No consistent pattern distinguished the two groups, and none of the differences between them was significant.

TABLE III.—*Proteolytic Activities (Mean ± S.E.) of Tumours from* 12 *Patients Showing Spread to Regional Lymph Nodes Compared with Values for Tumours from* 8 *Patients in whom no Evidence of Spread was Obtained.*

| | mg. Hb hydrolysed/hour/mg. nitrogen | | | | % Total activity in supernatant | |
|---|---|---|---|---|---|---|
| | Whole homogenate | Supernatant | Pellet | Nonidet increment | Before Nonidet | After Nonidet |
| pH 3·75 | | | | | | |
| Spread | 3·84±0·39 | 6·32±0·52 | 3·18±0·45 | 17·32±7·24 | 61·4±2·2 | 76·9±2·9 |
| No spread | 3·48±0·44 | 5·71±0·78 | 2·87±0·46 | 5·22±9·24 | 59·4±2·4 | 74·7±2·7 |
| pH 6·50 | | | | | | |
| Spread | 0·36±0·07 | 0·33±0·14 | 0·68±0·16 | 7·10±5·42 | 24·0±6·9 | 34·4±7·9 |
| No spread | 0·43±0·11 | 0·41±0·16 | 0·55±0·13 | 2·24±1·16 | 20·1±6·8 | 27·0±5·2 |

Since all the tumours studied were adenocarcinomata, it was possible that dilution of enzyme activity by protein-containing enzyme-free mucus might have been a factor in reducing the specific enzyme activity relative to protein, at least in the supernatant fraction of the tumours. To test this possibility, the tumours were classified on the basis of histological examination into three groups: well-differentiated, poorly-differentiated, and an intermediate group. The supernatant activities are presented in Table IV. If mucus production were an important factor in reducing supernatant enzyme activities of adenocarcinomata, poorly-differentiated tumours should have a higher specific activity in this fraction than well-differentiated tumours. In fact the opposite trend is apparent from the data, although the differences are not statistically significant due possibly to the small size of the various groups.

TABLE IV.—*Comparison of Proteolytic Activities (mg. Hb hydrolysed/hour/mg. Nitrogen) in Supernatant Fraction of Adenocarcinomata of Colon Classified as Well-differentiated (5 samples), Poorly-differentiated (5 Samples) and Intermediate (10 Samples). Results as Mean ± S.E.*

| | pH 3·75 | pH 6·50 |
|---|---|---|
| Well-differentiated | 7·42±1·41 | 0·38±0·16 |
| Intermediate | 6·01±1·32 | 0·38±0·12 |
| Poorly-differentiated | 4·78±1·59 | 0·27±0·13 |

### DISCUSSION

Normal human colonic epithelium contains proteolytic activity maximally active at pH 3·75 using Hb as substrate, and mainly associated with the supernatant fraction; detergent releases approximately half the activity originally associated with insoluble fractions (Goldberg *et al.*, 1969*a*). Evidence obtained from pH activity curves and study of purified cell fractions indicated the probable existence of a second enzyme, more intimately associated with insoluble fractions, which, while optimally active at acid pH, still has considerable activity at pH 6·50 (Goldberg *et al.*, 1969*a*). There does not seem to be a marked difference between normal and malignant epithelium in this respect, the pH activity curves obtained from both being similar (Fig. 1). This validates the comparison between

Fig. 1.—pH activity curves for proteolytic activity of fractions prepared from paired samples of uninvolved mucosa (solid circles) and adenocarcinoma (open circles). Buffers were 0·1 M acetate (pH 2·0 to 5·0) and 0·1 M phosphate (pH 5·5 to 7·5). Fractions are Homogenate (A), Supernatant (B) and Pellet (C). Activity of each normal fraction at pH 4·0 is taken as 100.

normal and malignant tissues at the two pH values chosen, a useful precaution when investigating an ill-defined enzyme or group of enzymes active over a wide pH range, since mouse ascites tumours have a pH profile for ribonuclease which differs from that of normal mouse tissues (Colter, Kuhn and Ellem, 1961), and the pH activity curve for ribonuclease in hyperplastic human thyroid tissue was not identical with that of the normal gland (Goldberg and Goudie, 1968).

The existence of a proteolytic enzyme similar to the cathepsin D of beef spleen (Press, Porter and Cebra, 1960) and of rabbit spleen (Lapresle and Webb, 1960, 1962) has been demonstrated in guinea-pig intestinal mucosa (Kregar, Turk and Lebez, 1967). Proteolytic enzymes of rat intestinal mucosa were found to be associated with lysosomes (Hsu and Tappel, 1964). It is unlikely that either of the two proteolytic activities measured in this work are lysosomal, despite the high specific activity of the Nonidet increment. Detergent did not increase the specific activity of the homogenate through activation of latent enzyme, but merely solubilised a small percentage (6%–15%) which happened to be present in the particles in a fully active form. It is likely that much of this activity originated in mitochondria which are known to be major sources of proteolytic enzymes (Alberti and Bartley, 1963, 1965, 1969). Mitochondria may be prepared in high yield from human colonic epithelium, but electron microscope studies on purified subcellular fractions show few organelles corresponding to lysosomes (Goldberg, Campbell and Roy, 1969); the paucity of lysosomes in human colonic epithelium was confirmed by electron microscope analysis of the fine structure of fresh surgical material (R. F. Macadam, personal communication).

Raised levels of proteolytic enzymes (Sylvén and Bois-Svenssen, 1965) and of dipeptidases (Wu and Bauer, 1963) have been found in rodent tumours. These observations are compatible with speculation that proteolytic enzymes may play a role in tumour invasiveness (Sylvén, 1968*a*, 1968*b*) and that release of lysosomal hydrolases might be concerned in cancer induction (Allison, 1966). Neither possibility finds support in the present work, since activity in all fractions was reduced in the cancers relative to two series of non-malignant tissues. We have previously shown reduced levels of proteolytic enzymes in colonic mucosa affected by non-malignant inflammatory diseases such as ulcerative colitis (Goldberg, McAllister and Roy, 1969*b*). Reduced levels of respiratory enzymes have been demonstrated by histochemical analysis of regenerating human colonic mucosa (Melnyk, Braucher and Kirsner, 1967). Certain oxidative enzymes, including succinic dehydrogenase, appear to be reduced in human colonic cancer (Wattenberg, 1959*a*, 1959*b*), and reduction in the aldolase content of human colonic tumours has also been reported (Dale, 1965). Although there is some basis in these reports for the view that loss of respiratory and degradative enzymes may be a non-specific response on the part of colonic mucosa to injury or disease, to regeneration or to increased cell turnover, the overall picture is by no means so consistent. For example, Dale (1965) found colonic cancer to have increased levels of lactate dehydrogenase and deoxyribonuclease II when enzyme activities were measured in relation to DNA content; activities of lactate dehydrogenase, malate dehydrogenase and enolase were raised in adenocarcinomata of the human colon relative to adjacent uninvolved tissue (Ames, Albaum and Antopol, 1964). Increased lactate dehydrogenase activity in human colonic cancer was also reported by Goldman, Kaplan and Hall (1964). In an extensive study, Shonk and colleagues found an increased content of most glycolytic enzymes in human colonic cancer, but reduced levels of phosphofructokinase, fructose-1,6-diphosphatase and alpha-glycerophosphate dehydrogenese were also noted (Shonk, Arison, Koven, Majima and Boxer, 1965). At the present time, it is therefore not possible to fit the observations in this report into a coherent and unified concept of metabolism in the cancer cell generally, or in colonic cancer as a specific entity.

The problem of cancer investigation in the human subject is rendered difficult by the lack of access to suitable control material. This problem has been emphasised in the present work through the wide differences existing between apparently normal tissue obtained at operation from patients with malignant and non-malignant bowel lesions. We cannot say with certainty whether the uninvolved tissue from cancer patients has less proteolytic activity than normal, or whether the uninvolved tissue from patients with inflammatory bowel diseases has higher activity than normal. The simple truth is that we do not know the characteristics of " normal " tissue. Since ulcerative colitis is associated with marked protein loss in the bowel (Steinfeld, Davidson and Gordon, 1957; Soergel and Ingelfinger,

1961), changes in serum proteins (Bicks, Kirsner and Palmer, 1959; De Dombal, 1968), and functional impairment of abdominal organs such as the liver (Vinnick, Kern and Corley, 1963) and pancreas (Ball, Baggenstoss and Bargen, 1950), in addition to the well known involvement of distant sites manifested by arthritis and occular changes, it is to be expected that subtle changes in cell metabolism manifested by altered enzyme levels might be present in morphologically normal adjacent bowel tissue. Similar considerations apply to cancer patients; indeed altered levels of certain enzymes of carbohydrate metabolism were found in the livers of patients with gastro-intestinal carcinomas without hepatic spread (Dacha, Catterina and Fornaini, 1963). The use of autopsy material has other dangers. Although most glycolytic enzymes have comparable activities in surgical and autopsy specimens, others such as phosphofructokinase rapidly lose activity in the latter (Shonk, Majima, Koven and Boxer, 1966). The effect of anoxia on acid hydrolases (De Duve and Beaufay, 1959) would render such material unsuitable for the study of the cytoplasmic distribution of proteolytic enzymes.

Although the biochemical features that distinguish malignant from normal tissue are a long way from being defined for the human subject, descriptive studies such as the present may contribute data on a small aspect which, when fitted into the pattern of future work, may one day permit this important distinction to be made.

### SUMMARY

Proteolytic activities determined by the hydrolysis of denatured haemoglobin at pH 3·75 and pH 6·50 have been measured in 20 adenocarcinomata of the human colon and the neighbouring uninvolved mucosa from the same patients. Reduced activities were found in soluble and insoluble cell fractions of the tumours at both pH values. The reduction could not be related to the degree of differentiation of the tumour or its invasiveness as gauged by spread to neighbouring lymph nodes. Proteolytic activities in the uninvolved mucosa of cancer patients were decreased compared with the levels found in ten samples of uninvolved mucosa from patients with inflammatory disease of the colon. The distribution of activities was studied before and after treatment of the homogenates with the detergent Nonidet P40. The percentage of the homogenate activity present in a soluble form was diminished at both pH values in the cancers both before and after Nonidet treatment, relative to uninvolved mucosa from non-cancer subjects; the samples of uninvolved mucosa from cancer subjects were intermediate between the above two groups in this respect. Although the reduced proteolytic activities of human colonic cancers seems established by this work, the characteristics of normal colonic mucosa are difficult to define in view of significant differences between the two control populations studied.

We are grateful to Professor A. W. Kay for his encouragement and guidance, and to Mr. D. B. Brown, Mr. D. A. Peebles-Brown, Mr. D. Clarke and Mr. A. B. Kerr who provided material for this investigation.

### REFERENCES

ALBERTI, K. G. M. M. AND BARTLEY, W.—(1963) *Biochem. J.*, **87**, 104.—(1965) *Biochem. J.*, **95**, 641.—(1969) *Biochem. J.*, **111**, 763.
ALLISON, A. C.—(1966) *Proc. Roy. Soc. Med.*, **59**, 867.

AMES, I. H., ALBAUM, H. G. AND ANTOPOL, W.—(1964) *Proc. Soc. exp. Biol. Med.*, **116**, 1013.
BALL, W. P., BAGGENSTOSS, A. H. AND BARGEN, J. A.—(1950) *Archs Path.*, **50**, 347.
BICKS, R. O., KIRSNER, J. B. AND PALMER, W. L.—(1959) *Gastroenterology*, **37**, 256.
COLTER, J. S., KUHN, J. AND ELLEM, K. A. O.—(1961) *Cancer Res.*, **21**, 48.
DACHA, U., CATTERINA, E. AND FORNAINI, G.—(1963) *Cancer, N.Y.*, **16**, 218.
DALE, R. A.—(1965) *Clinica chim. Acta*, **11**, 547.
DE DOMBAL, F. T.—(1968) *Gut*, **9**, 144.
DE DUVE, C. AND BEAUFAY, H.—(1959) *Biochem. J.*, **73**, 610.
GOLDBERG, D. M., CAMPBELL, R. AND ROY, A. D.—(1969) *Scand. J. Gastroenterology*, **4**, 217.
GOLDBERG, D. M. AND GOUDIE, R. B.—(1968) *Br. J. Cancer*, **22**, 220.
GOLDBERG, D. M., MCALLISTER, R. A. AND ROY, A. D.—(1969*a*) *Enzymologia*, **36**, 227.—(1969*b*) *Br. J. exp. Path.*, **50**, 241.
GOLDBERG, D. M. AND PITTS, J. F.—(1966) *Br. J. Cancer*, **20**, 729.
GOLDBERG, D. M., PITTS, J. F. AND AYRE, H. A.—(1967) *Br. J. Cancer*, **21**, 312.
GOLDMAN, R. D., KAPLAN, N. O. AND HALL, T. C.—(1964) *Cancer Res.*, **24**, 389.
HSU, L. AND TAPPEL, A. L.—(1964) *J. Cell Biol.*, **23**, 233.
KREGAR, I., TURK, V. AND LEBEZ, D.—(1967) *Enzymologia*, **33**, 80.
LAPRESLE, C. AND WEBB, T.—(1960) *Biochem. J.*, **76**, 538.—(1962) *Biochem. J.*, **84**, 455.
MELNYK, C. S., BRAUCHER, R. E. AND KIRSNER, J. B.—(1967) *Gastroenterology*, **52**, 985.
PRESS, E. M., PORTER, R. R. AND CEBRA, J.—(1960) *Biochem. J.*, **74**, 501.
PRUNTY, F. T. G., MCSWINEY, R. R. AND HAWKINS, J. B.—(1959) ‘A Laboratory manual of Chemical Pathology’. London (Pergamon Press). p. 165.
SHONK, C. E., ARISON, R. N., KOVEN, B. J., MAJIMA, H. AND BOXER, G. E.—(1965) *Cancer Res.*, **25**, 206.
SHONK, C. E., MAJIMA, H., KOVEN, B. J. AND BOXER, G. E.—(1966) *Cancer Res.*, **26**, 607.
SOERGEL, K. H. AND INGELFINGER, F. J.—(1961) *Gastroenterology*, **40**, 37.
STEINFELD, J. L., DAVIDSON, J. D. AND GORDON, R. S. Jr.—(1957) *J. clin. Invest.*, **36**, 931.
SYLVÈN, B.—(1968*a*) *Eur. J. Cancer*, **4**, 463.—(1968*b*) *Eur. J. Cancer*, **4**, 559.
SYLVÈN, B. AND BOIS-SVENSSEN, I.—(1965) *Cancer Res.*, **25**, 458.
VINNICK, I. E., KERN, F. Jr AND CORLEY, W. D.—(1963) *Gastroenterology*, **45**, 492.
WATTENBERG, L. W.—(1959*a*) *Am. J. Path.*, **35**, 113.—(1959*b*) *Cancer Res.*, **19**, 1118.
WU, C. AND BAUER, J. M.—(1963) *Cancer Res.*, **23**, 954.